# Monoclonal antibody with conformational specificity for a toxic conformer of amyloid β42 and its application toward the Alzheimer’s disease diagnosis

**DOI:** 10.1038/srep29038

**Published:** 2016-07-04

**Authors:** Kazuma Murakami, Maki Tokuda, Takashi Suzuki, Yumi Irie, Mizuho Hanaki, Naotaka Izuo, Yoko Monobe, Ken-ichi Akagi, Ryotaro Ishii, Harutsugu Tatebe, Takahiko Tokuda, Masahiro Maeda, Toshiaki Kume, Takahiko Shimizu, Kazuhiro Irie

**Affiliations:** 1 Division of Food Science and Biotechnology, Graduate School of Agriculture, Kyoto University, Kyoto, Japan; 2 Department of Advanced Aging Medicine, Chiba University Graduate School of Medicine, Chiba, Japan; 3 National Institute of Biomedical Innovation, Health and Nutrition, Osaka, Japan; 4 Department of Neurology, Kyoto Prefectural University of Medicine, Kyoto, Japan; 5 Department of Molecular Pathobiology of Brain Diseases, Kyoto Prefectural University of Medicine, Kyoto, Japan; 6 Immuno-Biological Laboratories Co, Ltd., Gunma, Japan; 7 Department of Pharmacology, Graduate School of Pharmaceutical Sciences, Kyoto University, Kyoto, Japan

## Abstract

Amyloid β-protein (Aβ42) oligomerization is an early event in Alzheimer’s disease (AD). Current diagnostic methods using sequence-specific antibodies against less toxic fibrillar and monomeric Aβ42 run the risk of overdiagnosis. Hence, conformation-specific antibodies against neurotoxic Aβ42 oligomers have garnered much attention for developing more accurate diagnostics. Antibody 24B3, highly specific for the toxic Aβ42 conformer that has a turn at Glu22 and Asp23, recognizes a putative Aβ42 dimer, which forms stable and neurotoxic oligomers more potently than the monomer. 24B3 significantly rescues Aβ42-induced neurotoxicity, whereas sequence-specific antibodies such as 4G8 and 82E1, which recognizes the N-terminus, do not. The ratio of toxic to total Aβ42 in the cerebrospinal fluid of AD patients is significantly higher than in control subjects as measured by sandwich ELISA using antibodies 24B3 and 82E1. Thus, 24B3 may be useful for AD diagnosis and therapy.

A hallmark of Alzheimer’s disease (AD) is amyloid deposition in senile plaques that consist mainly of 40- and 42-residue amyloid β-proteins (Aβ40 and Aβ42)^1^. These proteins are generated from Aβ-protein precursor by two proteases, β- and γ-secretases. Aβ aggregates (oligomerizes) through intermolecular β-sheet formation to exhibit neurotoxicity. The term “aggregation” in this context is defined as the change from Aβ monomers into amyloid fibrils *via* oligomers or protofibrils. Aβ42 plays a more important role in AD pathogenesis than Aβ40 because of its stronger ability to aggregate and show neurotoxicity^2^. Multiple lines of evidence have proposed that the soluble oligomeric assembly of Aβ is more exclusively involved in neuronal death and cognitive impairment than its insoluble fibrils and protofibrils^3^. The minimal unit of these oligomers, which have been divided into low molecular-weight oligomers (2–12-mer) and high molecular-weight oligomers (24–100-mer), is thought to be either a dimer^4^ or trimer^5^ (2 or 3 × *n*-mer). Aβ oligomer accumulation is among the earliest phenomena during the progression of AD pathology compared with other AD-related events such as hyperphosphorylation of tau protein, decreased hippocampal volume, and lowered glucose metabolism^6^. These highlight the significance of early and accurate diagnosis by targeting Aβ oligomer.

We have previously reported using solid-state NMR^7^ and systematic proline replacement^8^ that Aβ42 is held in equilibrium of the toxic conformer with a turn at Glu22 and Asp23, and the non-toxic one with a turn at Gly25 and Ser26 (Fig. 1a). In particular, the proportion of Aβ42 toxic conformer, in which residues Gln15~Ala21 and Val24~Ile32 are involved in forming an intermolecular parallel β-sheet of Aβ42 aggregates, may contribute to AD pathologies as well as cerebral amyloid angiopathy (CAA) such as Italian mutation^7^. Moreover, the C-terminal hydrophobic core derived from another turn at Gly38 and Val39, in addition to the intramolecular anti-parallel β-sheet (Met35~Gly37 and Val40~Ala42), accelerates the aggregation (oligomerization) of Aβ42^9^. The contribution of the C-terminal turn structure to the superior ability of Aβ42 to form oligomers has also been reported by other groups^10^,^11^,^12^. Based on this knowledge, we have proposed a dimer model of Aβ42 with the “toxic turn” at Glu22 and Aps23 and the C-terminal core, which is supposed to be a partial structure of toxic oligomers of Aβ42 (Fig. 1b)^9^.

We previously reported a conformation-targeted monoclonal antibody (11A1)^13^ against the toxic turn of Aβ42 generated by immunization with E22P-Aβ10–35, a minimum moiety for neurotoxicity including the toxic turn as a Pro-X corner (X: variable amino acid residue). 11A1 reacted with intracellular as well as extracellular Aβ depositions. In particular, the intracellular accumulation of Aβ could originate preferably from Aβ oligomers in AD brains^13^,^14^, AD-model mouse brains^14^,^15^, and neurons differentiated from induced pluripotent stem cells of AD patients^16^. However, the obvious reactivity of 11A1 with senile plaques (formed by extracellular Aβ accumulation) composed of fibrillar Aβ as well as monomeric Aβ hampers its application towards the diagnosis of AD. Indeed, because present diagnoses using sequence-specific antibodies against less-toxic fibrillar and Aβ42 monomer run a risk of false positives (approximately ~30%) of AD^17^,^18^, conformation-specific antibodies targeting neurotoxic Aβ42 oligomers have garnered much attention for developing more accurate diagnostic methods. Several conformation-specific antibodies such as anti-prefibrillar Aβ oligomers (OC)^19^, and anti-Aβ42-derived diffusible ligands (ADDLs) antibodies have been applied to AD diagnosis using cerebrospinal fluid (CSF). Although these attempts were likely successful in the limited cohort, almost all the target molecules of these antibodies could be Aβ oligomers that move into the fibrillar stage (on-pathway)^20^, so the possibility of false positives in the future trials using these antibodies cannot be excluded. Therefore, the development of monoclonal antibodies that specifically recognize toxic Aβ oligomers that exist as stable soluble intermediates (off-pathway) is an important challenge for selectively removing neurotoxic Aβ oligomers.

In this paper, we report a novel monoclonal antibody (named as 24B3) with greatly enhanced selectivity for the toxic Aβ42 conformer compared with 11A1, the first generation conformation-targeted antibody. Here, we describe the characterization of 24B3 by physicochemical and biochemical analyses. Notably, 24B3 recognizes the toxic oligomer model of Aβ42 (Fig. 1b) more preferentially than the corresponding monomer. Furthermore, 24B3 was applied to the diagnosis of AD using human CSF, as determined by a novel sandwich enzyme-linked immunosorbent assay (ELISA) that uses a combination of 24B3 and an anti-N-terminus-specific antibody, 82E1.

## Results

### Development of antibody 24B3 that specifically recognizes the toxic conformer of Aβ42

**Figure 1:**
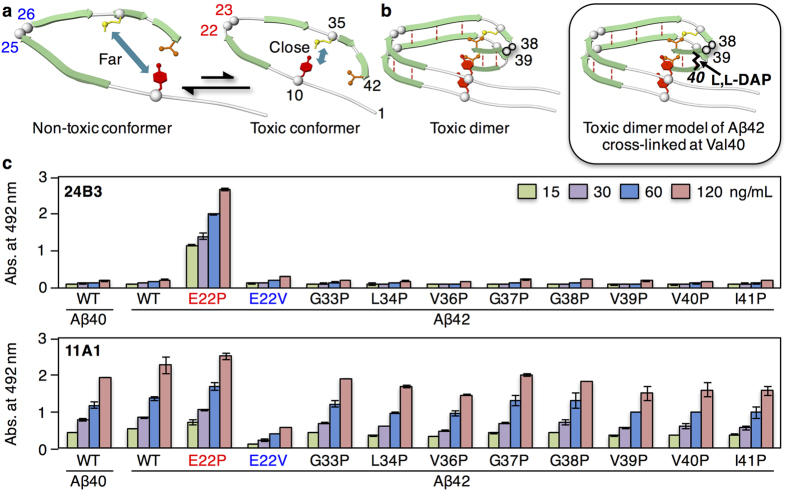
Development of antibody 24B3 that specifically recognizes a toxic conformer of Aβ42 with a turn at Glu22 and Asp23. (**a**) Aβ42 is held in equilibrium of the toxic conformer (*right*) with turn at positions 22 and 23, and the non-toxic one (*left*) with turn at positions 25 and 26. The proportion of toxic conformer of Aβ42 could be enhanced by E22P or E22K mutations^7^. (**b**) Dimerization of the toxic Aβ42 conformer that has a turn at Glu22 and Asp23 to induce close interaction between Tyr10 and Met35 for formation of the *S*-oxidized radical cation of Met35 and a C-terminal hydrophobic core for stabilization of radicals for long-lasting oxidative stress. Based on this hypothesis, a toxic oligomer model of Aβ42 covalently cross-linked using L,L-DAP at Val40 was designed. (**c**) Enzyme immunoassay using 24B3 and 11A1 (15, 30, 60, 120 ng/mL) to detect Aβ42 mutants (2.5 μg/well) substituted with proline at Glu22, Gly33, Leu34, Val36, Gly37, Gly38, Val39, Val40, and Ile41 together with Aβ40, Aβ42, and E22V-Aβ42, coated on plates.

To generate the antibody that specifically recognizes the “toxic turn” at Glu22 and Asp23 of Aβ42, the seven monoclones previously selected using E22P-Aβ10–35^13^ containing the toxic turn were re-evaluated in detail based on their ability to react with various Aβ42 mutants with a proline replacement mainly at C-terminal region^8^. In order to precisely evaluate the selectivity for toxic conformer of Aβ42, the concentration of clones used in this re-evaluation (15~120 ng/mL) was lower than that of the previous study^13^ (125~1,000 ng/mL). Enzyme immunoassay (EIA) identified a unique clone, named 24B3, as showing the strongest immunoreactivity with E22P-Aβ42 among the other mutants in a dose-dependent manner (Fig. 1c). The specificity of 24B3 for E22P-Aβ42 was much higher than that of 11A1. Moreover, 24B3 barely bound to E22V-Aβ42, in which valine is known as a turn breaker, while 11A1 reacted weakly with E22V-Aβ42 (Fig. 1c). These results suggest that 24B3 is more conformation-specific for the toxic Aβ42 conformer compared with 11A1 since the proportion of toxic conformer of Aβ42 could be enhanced by E22P mutation.

### Prevention of Aβ42-induced neurotoxicity by 24B3 occurs through sequestration of Aβ42 oligomers

**Figure 2:**
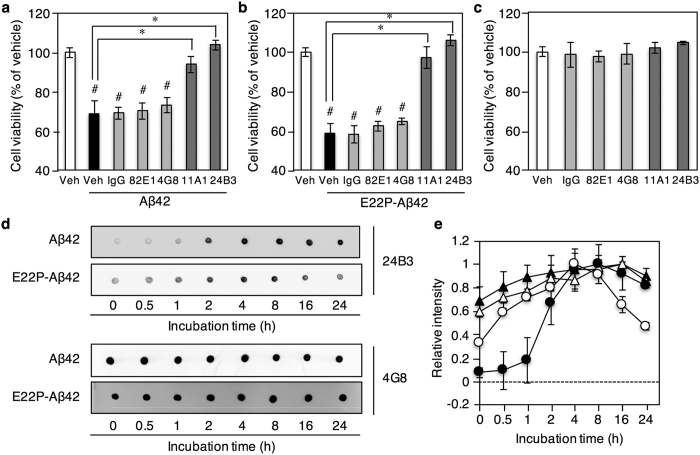
Prevention of Aβ42-induced neurotoxicity by sequestering Aβ42 oligomer using 24B3. (**a,b**) Neurotoxicity of (**a**) Aβ42 and (**b**) E22P-Aβ42 (1 μM) on SH-SY5Y cells, and effects of IgG, 82E1, 4G8, 11A1, and 24B3 (0.1 mg/mL) on Aβ-induced neurotoxicity after 48 h incubation, determined by MTT assay. (**c**) Effects of antibodies alone on the cell viability. ^#^*p* < 0.05 vs vehicle alone, *p < 0.05. Data are expressed as mean ± s.e.m. (*n* = 3). (**d,e**) Dot blotting for Aβ42 and E22P-Aβ42 using 24B3 and 4G8 for detection. Aβ solution (25 μM) was incubated for the indicated duration at 37 °C, and 1 μL of solution was spotted on the membrane at each time point. (**d**) Representative blots and (**e**) densitometric quantifications of immunoblot signals from triplicate samples are shown in (**d**), respectively. ●, 24B3 for Aβ42; ○, 24B3 for E22P-Aβ42; ▲, 4G8 for Aβ42; △, 4G8 for E22P-Aβ42.

To determine the effects of 24B3 on Aβ42-induced neurotoxicity, we performed the MTT assay on SH-SY5Y human neuroblastoma cells, a neuronal cell culture model. Not only 24B3 but also 11A1 significantly rescued the neurotoxicity induced by both Aβ42 and E22P-Aβ42 as a toxic conformer surrogate^8^, whereas neither 4G8 nor 82E1 (anti-N-terminus of Aβ42) could rescue (Fig. 2a,b). Notably, the protective effects of 24B3 were slightly higher than that of 11A1. All of these antibodies did not affect cell viability (Fig. 2c). Because we previously reported the neuroprotective potential of 11A1 on rat primary neurons^21^, the additional experiments were carried out. We then obtained similar results using primary neuronal cultures originated from rat (Supplementary Fig. 1). The slight inhibition of cytotoxicity by 11A1 was also observed with PC12 cells^13^. These observations are consistent with previous findings that A11 rescues Aβ42-induced toxicity in SH-SY5Y cells, whereas 6E10 does not^22^, raising the possibility that 24B3 may target the oligomeric species of Aβ responsible for neurotoxicity in a manner similar to A11 although the haptens of these antibodies are different from each other. These results suggest that conformation-specific antibodies may be more beneficial in treating AD pathology than sequence-specific antibodies, and that Aβ aggregates sequestered by 24B3 may contain oligomers formed by the toxic Aβ42 conformer.

Since both Aβ42 and E22P-Aβ42 induced neurotoxicity in a time-dependent manner^21^, we determined by dot blotting how 24B3 detects the toxic conformer of Aβ42. Regarding the immunoreactivity of 24B3, the relative intensity of E22P-Aβ42 gradually increased to a maximum after 4 h of incubation, whereas Aβ42 reacted more slowly than E22P-Aβ42 (Fig. 2d,e). It should be noted that the relative quantification of reactivity of 24B3 against Aβ42 was carried out under the longer exposure of blots due to its moderate affinity. It is noteworthy that the potent immunosignal of E22P-Aβ42 against 24B3 immediately after dissolution means more rapid formation of the toxic conformer by E22P-Aβ42 compared with Aβ42. In contrast, the relative intensity by 4G8 against Aβ42 was potent even after 0 h of incubation, and it remained almost constant up to 24 h (Fig. 2d,e). Under the longer exposure in the case of E22P-Aβ42 treated with 4G8 because of its weak affinity, the similar results to Aβ42 treated with 4G8 were obtained (Fig. 2d,e). These results suggest that the Aβ42 oligomers responsible for neurotoxicity can be attributed to those detected by 24B3, but not those detected by 4G8.

### Synthesis and characterization of the E22P-Aβ42 dimer as a toxic oligomer surrogate

We recently validated optically active L,L-diaminopimelic acid (DAP)^23^ as a useful linker near the intermolecular β-sheet region (Ala30) of E22P-Aβ40^24^. This strategy was applied to the synthesis of the E22P-Aβ42 dimer as a toxic oligomer model for Aβ42. The E22P mutation not only enhanced the neurotoxicity of Aβ42 ~10-fold^25^, but also increased the ratio of toxic conformation of Aβ42^7^. According to our proposed dimer model (Fig. 1b), Val40 in the C-terminal hydrophobic core was replaced with the linker. Solid-phase synthesis using Fmoc-L,L-DAP as a substitute for Val40 provided a sufficient amount of E22P-Aβ42 dimer (Fig. 1b) with high purity (6.0% yield, >98% purity, Supplementary Fig. 2).

**Figure 3:**
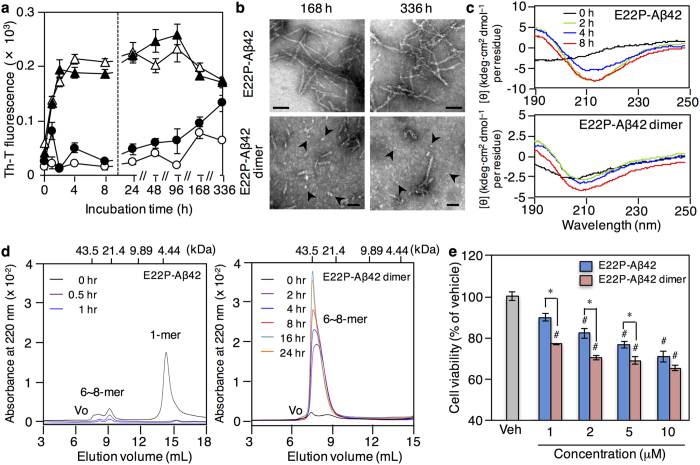
The E22P-Aβ42 dimer forms stable 6~8-mer associated with neurotoxicity. (**a**) Th-T assay result for each Aβ (25 μM) after incubation for the indicated duration at 37 °C. ●, the E22P-Aβ42 dimer; ○, the biotinylated E22P-Aβ42 dimer; ▲, E22P-Aβ42; △, biotinylated E22P-Aβ42. Data are expressed as mean ± s.e.m. (*n* = 8). (**b**) TEM analysis of Aβ aggregates formed from E22P-Aβ42 and E22P-Aβ42 dimer after incubation for the indicated duration at 37 °C. Arrowheads indicate oligomers. Scale bar = 50 nm. *Upper*, E22P-Aβ42; *lower*, the E22P-Aβ42 dimer. (**c**) Analysis of secondary structure of E22P-Aβ42 and the E22P-Aβ42 dimer (25 μM) after incubation for the indicated duration at 37 °C using CD spectrometry. (**d**) The ability of E22P-Aβ42 and E22P-Aβ42 dimer (25 μM) to form soluble oligomers was evaluated by size exclusion chromatography after incubation for the indicated duration at 37 °C. The peptide was detected by absorbance at 220 nm. Molecular marker sizes are shown in kDa. V_0_: void volume. (**e**) The viability of SH-SY5Y cells treated with either E22P-Aβ42 or E22P-Aβ42 dimer at the indicated concentration at 37 °C for 16 h. Data are expressed as the mean ± s.e.m. (*n* = 3). Absorbance obtained after adding vehicle (Veh: 0.1% NH_4_OH) was taken as 100%. ^#^*p* < 0.05 vs vehicle alone, *p < 0.05.

The aggregative ability of the E22P-Aβ42 dimer with a covalent linker at Val40 was evaluated using thioflavin-T (Th-T), a reagent that fluoresces when bound to Aβ aggregates, and transmission electron microscopy (TEM). E22P-Aβ42 aggregated with a lag time of ~4 h and a maximum fluorescence value after being incubated for 96 h (Fig. 3a), and its velocity for aggregation was higher than that of Aβ42, as previously reported^25^. In contrast, the fluorescence of the E22P-Aβ42 dimer remained almost unchanged even after a 96 h incubation (Fig. 3a). Given the moderate increase in the fluorescence after incubation for 168 and 336 h, these time-point samples were subjected to TEM analysis. As shown in Fig. 3b, we observed short and globular aggregates (oligomers) predominantly in the E22P-Aβ42 dimer, unlike the typical fibrils found in E22P-Aβ42.

Moreover, circular dichroism (CD) spectrometry was measured to analyze the secondary structure of the E22P-Aβ42 dimer. A positive peak at ~190 nm and a negative peak at ~210 nm began to increase gradually after dissolution, suggesting that a random structure transformed into a β-sheet architecture in the E22P-Aβ42 dimer in a manner similar to E22P-Aβ42 (Fig. 3c).

The ability of the E22P-Aβ42 dimer to form oligomers was further studied using size exclusion chromatography. As a control reference, the peaks corresponding to the monomer of E22P-Aβ42 and its 6~8-mer were observed immediately after dissolution, but these peaks disappeared almost completely after 1 h incubation (Fig. 3d). This observation suggests the formation of insoluble fibrillar aggregates of E22P-Aβ42, as observed in Fig. 3b. In contrast, the E22P-Aβ42 dimer formed stable oligomers (comprising 6~8-mers) during incubation for ~24 h even after dissolution (Fig. 3d). It should be noted that the peak of E22P-Aβ42 dimer did not appear to be observed even at the initial time point (Fig. 3d). Actually, we confirmed the presence of E22P-Aβ42 dimer in reverse-phase HPLC on ODS column (Supplementary Fig. 2b). Once dissolved in the PBS solution, the E22P-Aβ42 dimer could intrinsically form stable oligomers during elution in size exclusion chromatography. Alternatively, in the size exclusion chromatography using Superdex75 10/300GL column, E22P-Aβ42 dimer itself might not be able to give a sharp peak because of adopting various conformations. Similar phenomena were also observed in Aβ42 monomer using reverse-phase HPLC on ODS column under the acidic condition; Aβ42 was observed only as a broad peak^26^.

The neurotoxicity of the E22P-Aβ42 dimer was measured using the MTT assay on human neuroblastoma SH-SY5Y cells. After being incubated for 16 h when the stable oligomer of the E22P-Aβ42 dimer is suspected to be predominant, the viability of cells treated with the E22P-Aβ42 dimer was lower than that of E22P-Aβ42 at 1~10 μM, indicating that the E22P-Aβ42 dimer is more neurotoxic than E22P-Aβ42 (Fig. 3e). Overall, the E22P-Aβ42 dimer can form stable oligomers with a β-sheet structure, suggesting that the E22P-Aβ42 dimer is a suitable model for studying toxic Aβ42 oligomers. However, additional oligomer models such as trimer or tetramer should be examined to conclude which oligomeric species are toxic.

### Binding affinity of 24B3 for the E22P-Aβ42 dimer

**Figure 4:**
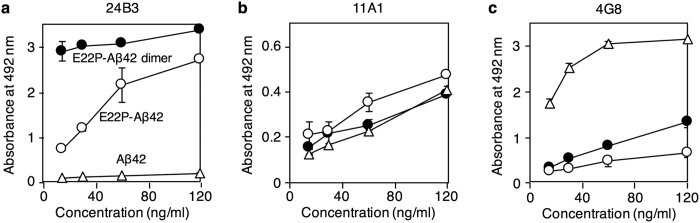
Specific recognition of E22P-Aβ42 dimer by 24B3. Enzyme immunoassay using (**a**) 24B3, (**b**) 11A1, and (**c**) 4G8 (15~120 ng/mL) to detect Aβ42, E22P-Aβ42, and the E22P-Aβ42 dimer (2.5 μg/well) coated on plates.

To investigate the potential of 24B3 to recognize toxic Aβ42 oligomers, we performed an EIA test. 24B3 showed stronger immunoreactivity against the E22P-Aβ42 dimer than E22P-Aβ42; its specificity for the E22P-Aβ42 dimer was much higher than that of 11A1 (Fig. 4a,b). In contrast, the relative affinity of 4G8 for the E22P-Aβ42 dimer was weak (Fig. 4c).

Surface plasmon resonance (SPR) was employed to evaluate the binding constant of 24B3 for Aβ, in which Aβ was immobilized on a sensor chip based on a biotin-streptavidin interaction. The biotinylation of Aβ42, E22P-Aβ42, and the E22P-Aβ42 dimer did not affect their aggregation profiles and neurotoxicity, respectively (Fig. 3a, Supplementary Fig. 3). 24B3 bound 6.5-fold more strongly to the E22P-Aβ42 dimer (*K*_D_ = 1.8 nM) than 11A1 (*K*_D_ = 10 nM) (Table 1, Supplementary Fig. 4). In addition, consistent with the EIA test (Fig. 4a,b), the binding affinity of 24B3 for E22P-Aβ42 (*K*_D_ = 3.1 nM) was ~8-fold higher than that of 11A1 for E22P-Aβ42 (*K*_D_ = 24 nM). Taken together with the lower binding affinity of 24B3 than 11A1 for Aβ42 (24B3: *K*_D_ > 100 nM, 11A1: *K*_D_ 15 nM), these results strongly suggest that 24B3 specifically recognizes toxic oligomers of Aβ rather than monomeric Aβ.

### Ratio of toxic conformer to total Aβ42 as a potential biomarker for AD pathology in CSF

**Figure 5:**
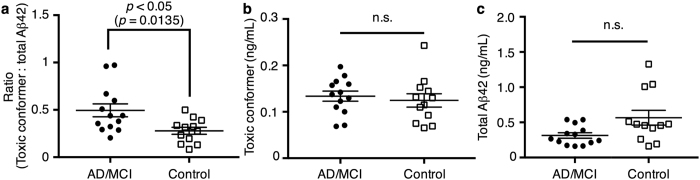
Comparing the ratio of toxic Aβ42 conformer to total Aβ42 in CSF between AD/MCI patients and age-matched individuals by sandwich ELISA. ELISA detection results: (**a**) ratio of toxic conformer of Aβ42 to total Aβ42, (**b**) toxic conformer of Aβ42, and (**c**) total Aβ42 in CSF of AD/MCI patients (*n* = 13) and age-matched controls (*n* = 12).

It is becoming important to identify and validate biomarkers in biological fluids for AD diagnosis, especially for prediction of patients with mild cognitive impairment (MCI) who will convert to AD. Given the potential of 24B3 as a specific probe for toxic Aβ42 oligomers, we developed a novel sandwich ELISA using a combination of anti-N-terminus antibody (82E1) for capture and 24B3 conjugated with horseradish peroxidase for detection. To determine if the ratio of toxic conformer to total Aβ42 is correlated with the AD pathology in the brain^7^, we performed ELISA on human CSF samples from 13 patients with AD/MCI and 12 age-matched controls (Table 2); we combined the patients with MCI and those with AD to compare with the age-matched controls, since all the MCI patients in this study were those with “MCI due to AD”^27^. Accordingly, the ratio of toxic conformer to total Aβ42 in AD/MCI patients was significantly higher than that in the age-matched controls (*p* = 0.0135, Fig. 5a). In contrast, the difference in total Aβ42 as one of the conventional biomarkers^28^ was not significant (*p* = 0.0863, Fig. 5c). Intriguingly, the absence of a significant difference in the amount of toxic Aβ42 conformer between AD/MCI and control groups (*p* = 0.4620, Fig. 5b) might be due to the disappearance of toxic oligomers by moving onto fibrillization of Aβ42 monomer. Indeed, the reduction of Aβ42 levels in CSF are originated from plaque formation by enhanced aggregation in the cerebral parenchyma as well as the disturbed clearance of Aβ from the cerebral parenchyma into CSF^28^. These results signify the proportion of toxic conformer of Aβ42 rather than its amounts. Considering its potential inaccuracy when to judge the diagnosis based on only the amount of total Aβ42^17^,^18^, the ratio of toxic conformer of Aβ42 to total Aβ42 could be a better substitute for precise diagnosis of AD.

## Discussion

Immunotherapy-mediated removal of Aβ aggregates is one of the first disease-modifying therapies for AD. However, several anti-Aβ antibodies recently tested in clinical trials failed^29^, possibly because they were evaluated in later stage AD patients, when neuronal cells have been irreversibly damaged. In this stage, the elimination of Aβ42 oligomers is no longer beneficial. Several ongoing immunotherapy trials involving patients diagnosed with preclinical AD or who carry a genetic mutation that induces AD symptoms may be more successful.

Our ideas are based on the concept that selective removal of toxic Aβ oligomers is important in immunotherapy since non-toxic Aβ plays a physiologically important role in synapse communication^30^ and regulation of glucose metabolism^31^. We developed the conformation-specific monoclonal antibody 24B3, which specifically recognizes the toxic conformer of Aβ42 in toxic oligomers (Fig. 4), and demonstrated a correlation between the ratio of toxic conformer to total Aβ42 and the pathology of AD (Fig. 5). Moreover, 24B3 suppressed significantly the Aβ-induced cytotoxicity *in vitro*, while sequence-specific antibodies such as 4G8 with weaker affinity for the E22P-Aβ42 dimer model did not (Figs 2 and 4). Several failures of passive immunization in clinical trials might be due to the insufficient affinity of sequence-specific antibodies^32^ for toxic Aβ42 conformers. Recent unexpected findings on the neuronal hyperactivity induced from immunotherapy using a sequence-specific antibody (3D6) reaffirm the significance of confirmation-specific antibody^33^. Otherwise, they might be ascribed to the unintended elimination of physiologically necessary non-toxic conformers of Aβ42^7^,^34^,^35^, though other reasons such as inadequate timing and period to treat with anti-Aβ antibodies have already been proposed^36^.

E22P-Aβ42 as a toxic conformer surrogate readily forms toxic oligomers to inhibit long term potentiation (LTP)^37^. Provided that Aβ42 oligomerization involves the formation of various transient or intransient assemblies, which are on- or off-pathway aggregation products, it is indispensable to develop an “off-pathway” Aβ42 oligomer model for developing anti-Aβ drugs with few side effects. To the best of our knowledge, this is the first report on the generation and characterization of an Aβ42 dimer model, which is connected at the C-terminal hydrophobic region. Practical synthesis of Aβ dimers has thus far been limited to Aβ40 dimers^4^,^23^,^38^, partly due to the intrinsic and potent ability of Aβ42 to aggregate during synthesis and preparation. Cross-linkage within this region has never been achieved in spite of its significance in oligomerization^10^,^11^,^12^. Although one study has reported the synthesis of a dityrosine cross-linked Aβ42 dimer at Tyr-10^38^, its biological activity was not tested due to insufficient yield. Other oligomer models with high molecular weight such as ADDLs (~24-mer)^39^ are also considered off-pathway aggregates. Dimers, trimers, and tetramers of Aβ42, as prepared by photo-induced cross-linking of unmodified protein (PICUP) technology using 2,2′-bipyridyl-dichlororuthenium(II) hexahydrate as a catalyst, were mainly bound covalently at Tyr10^40^, although it seems difficult to isolate these oligomers in a pure form. Recently, unique synthesis of N-terminal-tethered triple Aβ fragment (Aβ25–35) has been reported^41^ as a trimeric model, which may be another minimal unit of toxic oligomers (2 or 3 × *n*-mer) of Aβ42. When we take into account these models as candidates of toxic oligomers, further investigation will be required to clarify the significance *in vivo*, though the pathology of the genetically modified mice to produce Aβ dimer cross-linked at Ser8 in N-terminal region was recently reported^42^.

We identified 24B3 based on the ability to bind the conformer possessing the turn structure at residues 22 and 23 using some proline-substituted mainly at C-terminal core region mutants of Aβ42 (Fig. 1). Our findings indicate that 24B3 could be more favorable to the toxic conformer of Aβ42 with the turn at residues 22 and 23 than to 11A1. It should be noted that 11A1 may bind various conformers with turns at other residues, even though 11A1 is not proline-specific like 24B3. The enhanced reactivity of 24B3 with the E22P-Aβ42 dimer compared with E22P-Aβ42 (Fig. 4) suggests the stabilization of the toxic Aβ42 oligomer by formation of the C-terminal core and intermolecular β-sheet. Furthermore, the time-dependent reactivity of 24B3 towards Aβ42 in solution (Fig. 2) reflects the amount of toxic Aβ42 oligomers formed during incubation.

In previously reported sandwich ELISAs for Aβ oligomers^43^, the same anti-Aβ antibodies were used for both capture and detection based on the idea that oligomers have multiple reactive sites. However, it is likely to be difficult to discriminate toxic Aβ oligomers from less-toxic fibrillar Aβ aggregates using such ELISAs, and their validity has thus been questioned^44^. It is reasonable to use the conformation-specific antibody for detection after capturing total Aβ using 82E1 in sandwich ELISA. Decreased Aβ42, increased total tau, and increased phosphorylation of tau, are currently the most accepted biomarkers for diagnosing AD (probable, possible, or definite AD)^28^. There have also been intensive studies on other biomarkers such as Aβ-related or tau-related molecules in order to increase diagnostic validity by modifying sensitivity and specificity. However, outliers largely affect the validity in these conventional biomarkers, and there have been no studies on the qualitative difference between various Aβ conformers in spite of accumulating structural studies of Aβ. Our group proposed the significance of the ratio of the toxic conformer to total Aβ42 in AD pathogenesis^7^ using solid-state NMR analysis, which demonstrated that a ~2-fold increase in the ratio of toxic conformer of E22K-Aβ42 to wild-type Aβ42 can in part explain the extensive aggregative ability and neurotoxicity of E22K-Aβ42^25^. Although the ratio of Aβ42 to Aβ40 has been suggested as another biomarker^45^, such a biomarker might correlate more preferably with the amount of senile plaque containing less-toxic fibrils rather than with the amount of toxic oligomers.

In summary, the target of a novel conformation-specific monoclonal antibody 24B3 could be oligomers that include the toxic conformer of Aβ42, and the ratio of toxic conformer to total Aβ42 could be an alternative evaluation criterion toward accurate diagnosis of AD. Although the analysis with a larger number of CSF samples is indispensable for further validation, the development of a less invasive test using plasma is an attractive goal for the future. 24B3 could be promising as a diagnostic tool of AD because of its superior affinity for toxic oligomers consisting of toxic conformation of Aβ42, which is likely to be formed at an earlier stage before AD symptoms. Moreover, it could be applied to therapeutics during the early stages of AD.

## Methods

### Synthesis of biotinylated E22P-Aβ42

Biotin-E22P-Aβ42 was synthesized in a stepwise fashion on 0.1 mmol of preloaded Fmoc-L-Ala-PEG-PS resin (Applied Biosystems) by Pioneer^TM^ using the Fmoc method, as previously described^25^. Biotin was added as the final residue. Briefly, after the completion of chain elongation and cleavage from the resin, the crude peptide was precipitated using diethylether, followed by purification using HPLC on Develosil ODS UG-5 column (20 mm i.d. × 150 mm; YMC) with elution at 8.0 mL/min by an 80 min linear gradient of 10–50% CH_3_CN containing 0.1% NH_4_OH. Lyophilization yielded a pure peptide, the purity of which was confirmed by HPLC (>98%, 9.9% yield). The molecular weight of biotin-E22P-Aβ42 was confirmed by liquid chromatography-mass spectrometry, followed by deconvolution (LC-MS; Acquity UPLC system H-class with Xevo G2-S, Waters); *m*/*z*, calculated: 4709.44; observed: 4708.95 [MH]^+^ (Supplementary Fig. 2a).

### Synthesis of the E22P-Aβ42 dimer and its biotinylated derivative

Fmoc-L,L-DAP was either synthesized as described previously^24^ or purchased from Sigma. The E22P-Aβ42 dimer (Fig. 1b) or the biotin-E22P-Aβ42 dimer was synthesized as basically described above, except that a pseudoproline dipeptide, Fmoc-Gly-L-Ser(ψMe,Mepro)-OH (Novabiochem), was utilized at the positions Gly25 and Ser26. Val40 was replaced with L,L-DAP as a cross linker. Regarding the E22P-Aβ42 dimer, the purification using HPLC on YMC-Pack ODS-A column (20 mm i.d. × 150 mm; YMC) with elution at 8.0 mL/min by a 70 min linear gradient of 20–60% CH_3_CN containing 0.1% trifluoroacetic acid was carried out. Subsequent purification was performed using a YMC-Pack PROTEIN RP column (20 mm i.d. × 150 mm; YMC) with elution at 8.0 mL/min by an 80 min linear gradient of 20–60% CH_3_CN containing 0.1% trifluoroacetic acid. Lyophilization gave a pure peptide, the purity of which was confirmed by HPLC (>98%, 6.0% yield). The molecular weight of the E22P-Aβ42 dimer was confirmed by LC-MS, followed by deconvolution; *m*/*z*, calculated: 8920.16; observed: 8920.29 [M]^+^ (Supplementary Fig. 2b).

Regarding the biotin-E22P-Aβ42 dimer, the purification using HPLC on YMC-Pack ODS-A column (20 mm i.d. × 150 mm; YMC) with elution at 8.0 mL/min by an 80 min curve gradient (curve 7 in Waters M600 system) of 30–60% CH_3_CN containing 0.1% trifluoroacetic acid was carried out. Subsequent purification was performed using an XBridge BEH C18 Prep column (19 mm i.d. × 150 mm; Waters) with elution at 8.0 mL/min by an 80 min linear gradient of 10–50% CH_3_CN containing 0.1% NH_4_OH. Lyophilization gave a pure peptide, the purity of which was confirmed by HPLC (>98%, 3.4% yield). The molecular weight of the biotin-E22P-Aβ42 dimer was confirmed by LC-MS, followed by deconvolution; *m*/*z*, calculated: 9372.78; observed: 9372.92 [M]^+^ (Supplementary Fig. 2c).

### HFIP treatment of Aβ

For treatment with 1,1,1,3,3,3-hexafluoro-2-propanol (HFIP; Wako), each Aβ was dissolved in HFIP at 1 mg/mL. After incubation at room temperature for 30 min, the solution was sonicated for 5 min, and dried *in vacuo*. The resultant film of Aβ was stored at −80 °C until use.

### Thioflavin-T (Th-T) assay

The aggregative ability of each Aβ was evaluated with a previously described thioflavin-T (Th-T; Sigma) fluorescence assay^25^. Aβ was dissolved in 0.1% NH_4_OH at 250 μM, followed by 10-fold dilution with phosphate buffered saline (PBS; 50 mM sodium phosphate, and 100 mM NaCl, pH 7.4) to a final concentration of 25 μM. After incubating at 37 °C for the desired period, 2.5 μL of the reaction solution was added to 250 μL of 5.0 μM Th-T in 5.0 mM Gly-NaOH (pH 8.5), followed by the measurement of fluorescence at 430 nm excitation and 485 nm emission using a microplate reader (Fluoroskan Ascent; Thermo Scientific).

### Transmission electron microscopy (TEM)

The Aβ aggregates after incubation for the desired period in the Th-T assay were examined under a H-7650 electron microscope (Hitachi). The experimental procedure has been described elsewhere^25^. After the supernatant was removed from the pellets, the resultant aggregates were then suspended in water (100 μL) by gentle vortex mixing, and centrifuged at 6,600 rpm for 1 min. These suspensions were applied to a 200 mesh Formvar-coated copper grid (Nissin EM), and allowed to dry in air for 5 min after being negatively stained for several seconds with 2% uranyl acetate and subsequently subjected to microscopy.

### Circular dichroism (CD) spectrometry

The secondary structure of the Aβ dimer was estimated by CD spectrometry (J-805; JASCO) using a 0.1 mm quartz cell, as described elsewhere^37^. The Aβ solution (25 μM) prepared above was incubated at 37 °C. An aliquot was loaded into the quartz cell, and CD spectra were recorded at 190–260 nm. The spectra of Aβ are shown after subtraction of the spectrum for the vehicle alone.

### Size exclusion chromatography

The Aβ solution (25 μM) was incubated at 37 °C. After the solution was collected periodically and centrifuged at 17,860 × *g* at 4 °C for 10 min, the supernatant was analyzed by size exclusion chromatography on the Superdex75 10/300GL column (10 mm i.d. × 300 mm; GE Healthcare) with elution at 0.6 mL/min by filtered- and degassed-PBS (pH 7.4), attached to a Waters LC system with a 2489 UV/Visible detector and 1525 binary HPLC pump controlled by Empower^TM^3 software (Waters), as described elsewhere^24^. The peptide was detected by absorbance at 220 nm. Calibration curves of size exclusion columns were constructed using dextran standards (Mp: mean peak molecular weight, 43500, 21400, 9890, 4440) (Sigma) together with Blur dextran 2000 (GE Healthcare) as an indicator of the void volume (V_0_).

### MTT assay on SH-SY5Y cells

Human neuroblastoma, SH-SY5Y cells, maintained in a 1:1 mixture of Eagle’s minimum essential medium (Wako) and Ham’s F12 medium (Wako) containing 10% fetal bovine serum (Biological Industries), were used as a neuronal cell model to estimate the neurotoxicity of each Aβ with a slight modification to the previously described method^24^. In brief, each Aβ was dissolved in 0.1% NH_4_OH to generate a 10X stock solution. The resultant peptide solution (10 μL) was diluted with 0.1% NH_4_OH to appropriate final concentrations in medium before being added to 100 μL of the culture medium of near-confluent cells (10^4^ cells/well) after one or two overnight incubation. In the case to test the effect of antibodies on the cells, the culture medium was replaced with fresh medium containing pre-incubated (30 min) Aβ solution with antibodies. After being treated at 37 °C for 16 or 48 h, 10 μL of 5 mg/mL MTT (Sigma) was added to cells, followed by incubation for 4 h at 37 °C. After removing the medium, 100 μL cell lysis buffer (10% SDS, 0.01 M NH_4_Cl) was subsequently added to the cells. The resulting cell lysate was subsequently incubated overnight in the dark at room temperature before absorbance measurements were made at 595 nm with a microplate reader (MultiScan JX; Thermo Scientific). Absorbance obtained by the addition of vehicle (0.1% NH_4_OH) was taken as 100%.

### MTT assay on rat primary neurons

Animals were treated in accordance with guidelines by the Kyoto University Animal Experimentation Committee and guidelines by The Japanese Pharmacological Society. This study was approved by Kyoto University Animal Experimentation Committee. Neuronal cultures were obtained from the cerebral cortices of fetal Wistar rats (Nihon SLC) at 17–19 days of gestation as described previously^21^. Cultures were maintained in Neurobasal medium with 2% B-27 supplement, 25 μM sodium glutamate, and 0.5 mM L-glutamine at 37 °C in a humidified atmosphere of 5% CO_2_. After 4 days in culture, medium was replaced with sodium glutamate-free Neurobasal medium. Only mature cultures (8~12 days *in vitro*) were used for the experiments. In all experiments, B-27 supplement without antioxidants was utilized during the treatment of Aβ42 as described previously^21^.

Neurotoxicity was assessed by MTT assay according to the previously reported protocol^21^. After 30 min of pre-incubation on ice for Aβ42 solution (10 μM) in 0.1% NH_4_OH, followed by 10-fold dilution with Neurobasal medium, the medium containing Aβ was added to the cell culture for replacement. After incubation of Aβ42 at 37 °C for 96 h, the culture medium was replaced with medium containing 0.5 mg/mL MTT, and cells were incubated for 30 min at 37 °C. 2-Propanol was added to lyse the cells, and absorbance was measured at 595 nm with an absorption spectrometer (microplate reader model 680, Bio-rad). The medium of vehicle treatment for each experiment contained 0.01% NH_4_OH. The absorbance obtained by the addition of vehicle (0.1% NH_4_OH) was taken as 100%.

### Enzyme immunoassay (EIA)

A 96-well Maxisorp plate (Nunc) was incubated with each Aβ (2.5 μg/well) dissolved in 50 mM sodium carbonate for 2 h at room temperature, followed by treatment for blocking with 5% bovine serum albumin at 4 °C overnight, as described previously^13^. Briefly, after incubation with each clone obtained in the previous work^13^ for 1 h at room temperature, the plate was treated with a horseradish peroxidase-coupled anti-mouse IgG antibody (IBL), and quantified using *o*-phenylenediamine dihydrochloride substrate (Sigma) before measurements at 492 nm with a microplate reader (MultiScan JX; Thermo Scientific).

### Surface plasmon resonance (SPR)

Binding affinity tests were performed using a BIAcore X100 biosensor (GE Healthcare), as previously described^13^. In brief, sensor chip SA, to which streptavidin was anchored, was preconditioned by running HBS-EP buffer (GE Healthcare). Each HFIP-treated biotinylated Aβ dissolved in HBS-EP buffer (1 nM) was immobilized on the chip according to the manufacturer’s protocol. The antibody was dissolved in HBS-EP buffer and injected over the chip-immobilized Aβ at a flow rate of 5 μL/min. Either 20 mM Gly-HCl buffer (pH 2.0) or 5 M guanidine hydrochloride was used as the regeneration buffer. The values of response units (RU) obtained from a sample cell minus the RU obtained from a reference cell were used for analysis. Association and dissociation data were collected with flowing running buffer for 180 s and 360 s, respectively. The association and dissociation rate constants, and dissociation constant (*k*_a_, *k*_d_, and *K*_D_) were calculated using a serial dilution series of antibody concentrations according to BIAevaluation 3.1 software (GE Healthcare).

### Dot blotting

One microliter of each Aβ solution (25 μM) was applied to a nitrocellulose membrane after incubation at 37 °C (0.2 μm pore size; Bio-rad) as previously described^21^. After blocking in 5% non-fat milk dissolved in Tris-buffered saline containing 0.1% Tween-20 overnight at 4 °C, the membrane was treated with 24B3 or 4G8 (0.5 μg/mL) for 1 h at room temperature before being incubated with secondary antibody. Development was performed with enhanced chemiluminescence and quantified using LAS-4000 (Fujifilm). ImageJ 1.42 (NIH) software was used to quantify the blots.

### Subjects and collection of CSF samples

This study was conducted in accordance with the principles of Helsinki Declaration. The study was approved by the University Ethics Committee of Kyoto Prefectural University of Medicine. All subjects provided written informed consent to participate in the study. We collected CSF samples from 13 patients [aged 68~84 (mean ± SD, 77.1 ± 5.6) yr] with clinically diagnosed AD (*n* = 8) or MCI (*n* = 5), and 12 age-matched control subjects [aged 61~84 (mean ± SD, 72.3 ± 5.8) yr; see Table 2 for characteristics of study participants]. At the time of diagnosis, a full clinical history was taken, and physical and neurological examinations, Mini-Mental State Examination (MMSE), routine blood analyses, and magnetic resonance imaging (MRI) of the brain were performed for all subjects. The patients with AD met the criteria for probable AD defined in the diagnostic and research criteria established by the National Institute on Aging (NIA) and the Alzheimer’s Association (AA)^46^. All MCI patients were non-demented, not fulfilling the criteria for probable AD dementia^46^, but met the criteria of “MCI due to AD” defined by NIA and AA^27^. There were no significant differences in age between the AD/MCI group and the control group. None of the control subjects had memory complaints or any other cognitive symptoms.

Fresh CSF samples were obtained from the enrolled subjects and then immediately stored at −80 °C until used for immunoassays. All lumbar punctures were performed in the early morning to exclude the effects of daily fluctuation in the levels of Aβ in CSF^47^.

### Enzyme-linked immunosorbent assay (ELISA)

Microtiter plates (96 wells) were coated with 100 μL/well of 50 mM sodium carbonate containing 82E1 (IBL) and allowed to adhere overnight at 4 °C. Plates were washed with PBS and blocked for overnight at 4 °C with 200 μL/well of 1% (w/v) bovine serum albumin in PBS containing 0.05% NaN_3_. After two washes with PBS containing 0.02% Tween-20 (PBS-T), 100 μL of CSF was serially diluted in 1% bovine serum albumin in PBS-T before being added in triplicate to wells before incubation overnight at 4 °C. After four washes with PBS-T, each well was treated with 100 μL of horseradish peroxidase-conjugated 24B3 for 1 h at 4 °C. ELISA signals were detected by chemiluminescence using an enhanced chemiluminescent substrate (SuperSignal ELISA Femto Maximum Sensitivity, Thermo Scientific), and then measured with a microplate luminometer (SpectraMax Pro, Molecular Devices).

Synthetic E22P-Aβ40 dimer^24^ was used as a standard protein because the E22P-Aβ40 dimer also containing the toxic turn at Glu22 and Asp23 was more stable than the E22P-Aβ42 dimer. The concentration of the E22P-Aβ40 dimer was determined using the Bradford assay (Bio-Rad). The amount of total Aβ42 in CSF was determined by sandwich ELISA with a human β Amyloid ELISA Kit of Aβ42 [Cat# 27711, human amyloid β (1–42) including (X-42)] (IBL) according to the manufacturer’s protocols.

### Statistical analysis

All data are presented as mean ± s.e.m. The differences were analyzed with one-way analysis of variance (ANOVA) followed by Bonferroni’s test or unpaired Student’s *t*-test. These tests were implemented within GraphPad Prism software (version 5.0d). *p* values <0.05 were considered significant.

## Additional Information

**How to cite this article**: Murakami, K. *et al*. Monoclonal antibody with conformational specificity for a toxic conformer of amyloid β42 and its application toward the Alzheimer’s disease diagnosis. *Sci. Rep.* **6**, 29038; doi: 10.1038/srep29038 (2016).

## Supplementary Material

Supplementary Information

## Figures and Tables

**Table 1:** Dissociation constant (*K*_D_) together with association (*k*_a_) and dissociation (*k*_d_) rate constants of 24B3 and 11A1 for Aβ derivatives.

Antibody	Immobilized Aβ	*k*_a_ (M^−1^ s^−1^)	*k*_d_ (s^−1^)	*K*_D_(nM)
24B3	Aβ42	>5.0 × 10^2^	5.0 × 10^−5^	>100
	E22P-Aβ42	2.3 (0.015)* × 10^5^	7.2 (0.12) × 10^−4^	3.1^a^** (0.072)
	E22P-Aβ42 dimer	2.3 (0.017) × 10^5^	4.2 (0.25) × 10^−4^	1.8^b^ (0.092)
11A1	Aβ42	3.3 (0.12) × 10^4^	4.8 (0.23) × 10^−4^	15^c^ (0.93)
	E22P-Aβ42	4.0 (0.040) × 10^4^	9.5 (0.27) × 10^−4^	24^d^ (0.81)
	E22P-Aβ42 dimer	9.2 (0.98) × 10^2^	9.4 (1.6) × 10^−6^	10^e^ (0.94)

^*^The values in the parentheses indicate standard deviation from triplicate experiments.

^**^The different letters show significant differences.

**Table 2:** Summary of clinical diagnosis and mental state*.

Case	Age	Sex	MMSE	Clinical diagnosis
1	68	M	24	AD
2	69	F	18	AD
3	77	F	21	AD
4	82	F	18	AD
5	75	F	18	MCI
6	84	F	21	MCI
7	78	M	13	AD
8	81	M	23	MCI
9	75	M	N/A	AD
10	82	F	23	MCI
11	84	M	22	AD
12	68	M	20	AD
13	79	F	28	MCI
14	58	M		Control
15	73	F		Control
16	70	F		Control
17	71	F		Control
18	68	F		Control
19	81	F		Control
20	77	F		Control
21	69	F		Control
22	71	F		Control
23	77	M		Control
24	78	M		Control
25	74	M		Control

^*^MMSE, mini-mental state examination; AD, Alzheimer’s disease; MCI, mild cognitive impairment; N/A, not available.
