# Supplementary material for: Monoclonal antibody with conformational specificity for a toxic conformer of amyloid β42 and its application toward the Alzheimer’s disease diagnosis
Source: Sci Rep. 2016 Jul 4;6:29038. doi: 10.1038/srep29038 (PMC4931470; doi:10.1038/srep29038)
Supplement: Supplementary Information [file srep29038-s1.pdf]

Supplementary Information

Monoclonal antibody with conformational specificity for a toxic conformer of amyloid β42 and its application toward the Alzheimer's disease diagnosis

Kazuma Murakami[1], Maki Tokuda[1], Takashi Suzuki[1], Yumi Irie[1], Mizuho Hanaki[1], Naotaka Izuo[2], Yoko Monobe[3], Ken-ichi Akagi[3], Ryotaro Ishii[4], Harutsugu Tatebe[4], Takahiko Tokuda[4,5], Masahiro Maeda[6], Toshiaki Kume[7], Takahiko Shimizu[2], and Kazuhiro Irie[1,*]

[1]*Division of Food Science and Biotechnology, Graduate School of Agriculture, Kyoto University, Kyoto, Japan.*
[2]*Department of Advanced Aging Medicine, Chiba University Graduate School of Medicine, Chiba, Japan.*
[3]*National Institute of Biomedical Innovation, Health and Nutrition, Osaka, Japan.*
[4]*Department of Neurology, Kyoto Prefectural University of Medicine, Kyoto, Japan.*
[5]*Department of Molecular Pathobiology of Brain Diseases, Kyoto Prefectural University of Medicine, Kyoto, Japan.*
[6]*Immuno-Biological Laboratories Co, Ltd., Gunma, Japan.*
[7]*Department of Pharmacology, Graduate School of Pharmaceutical Sciences, Kyoto University, Kyoto, Japan.*

*Corresponding Author
Kazuhiro Irie, Ph.D., e-mail: irie@kais.kyoto-u.ac.jp.

[Contents]

Supplementary Figure 1~4

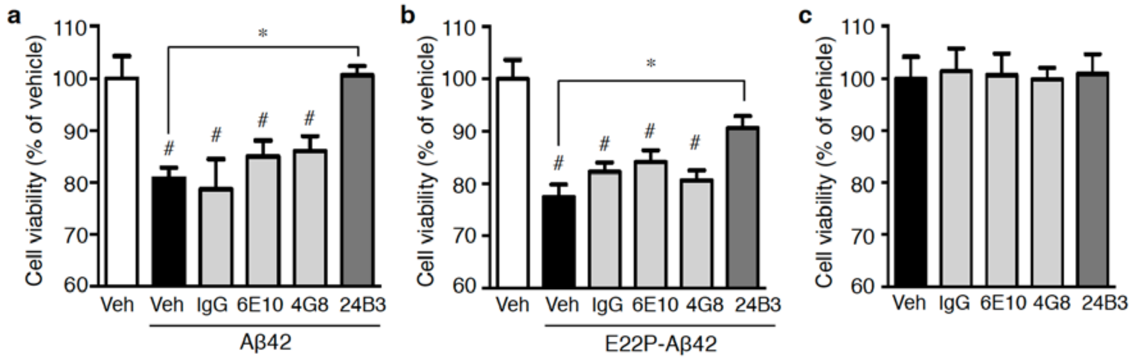

Supplementary Figure 1. Prevention of Aβ42-induced neurotoxicity by 24B3. (**a, b**) Neurotoxicity of (**a**) Aβ42 and (**b**) E22P-Aβ42 (1 μM) on rat primary neurons, and their effects by IgG, 6E10, 4G8, and 24B3 (0.1 mg/mL) on the Aβ-induced neurotoxicity after 96-hr incubation, evaluated by MTT assay. (**c**) Effects of antibodies alone on the cell viability. *$p < 0.05$ vs vehicle, #$p < 0.05$ vs vehicle without Aβ. Data are expressed as mean ± s.e.m. ($n = 3$).

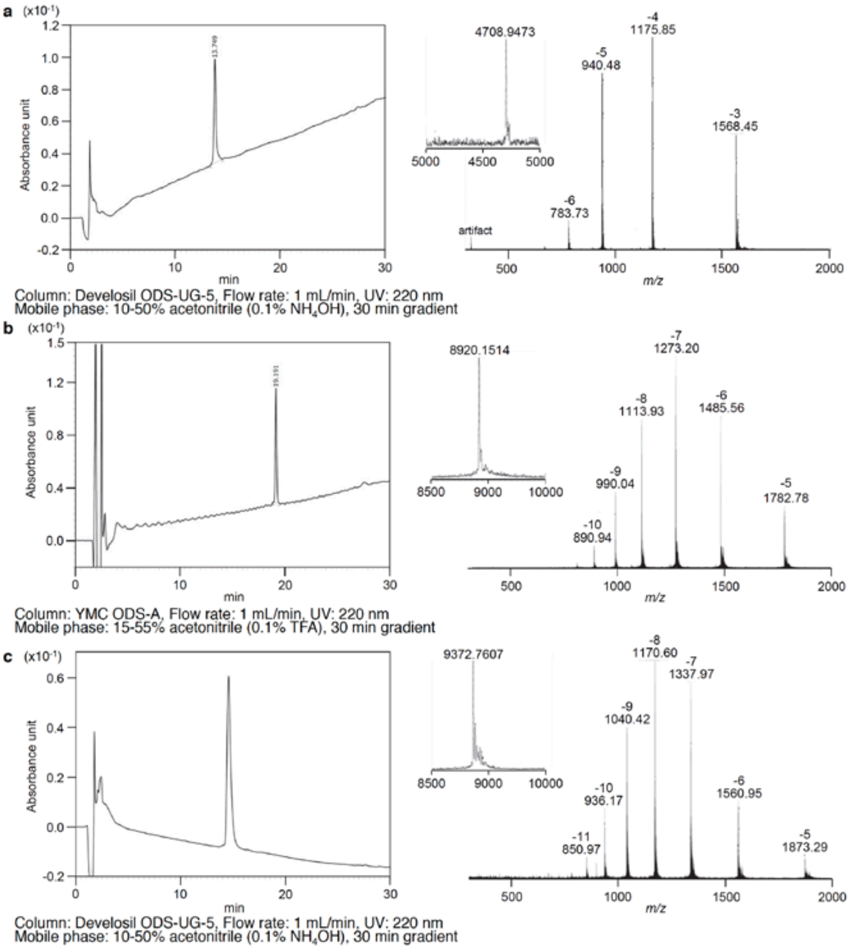

Supplementary Figure 2. HPLC and LC-MS data with deconvolution. (**a**) Biotin-E22P-Aβ42, (**b**) E22P-Aβ42 dimer, and (**c**) biotin-E22P-Aβ42 dimer.

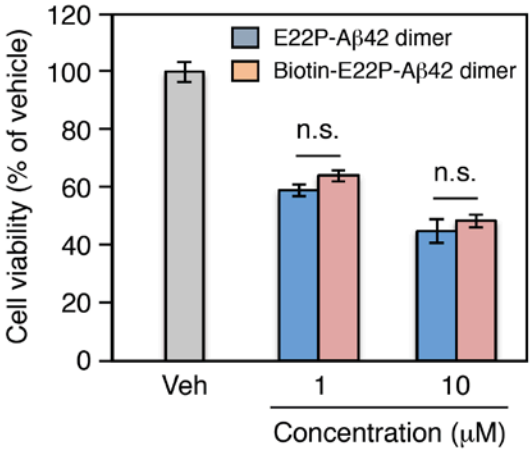

Supplementary Figure 3. Effects of biotinylation of the E22P-Aβ42 dimer on its neurotoxicity by MTT assay. Neurotoxicity of E22P-Aβ42 dimer and biotin-E22P-Aβ42 dimer (1 and 10 μM) on SH-SY5Y cells. Data are expressed as mean ± s.e.m. ($n = 3$). n.s., not significant.

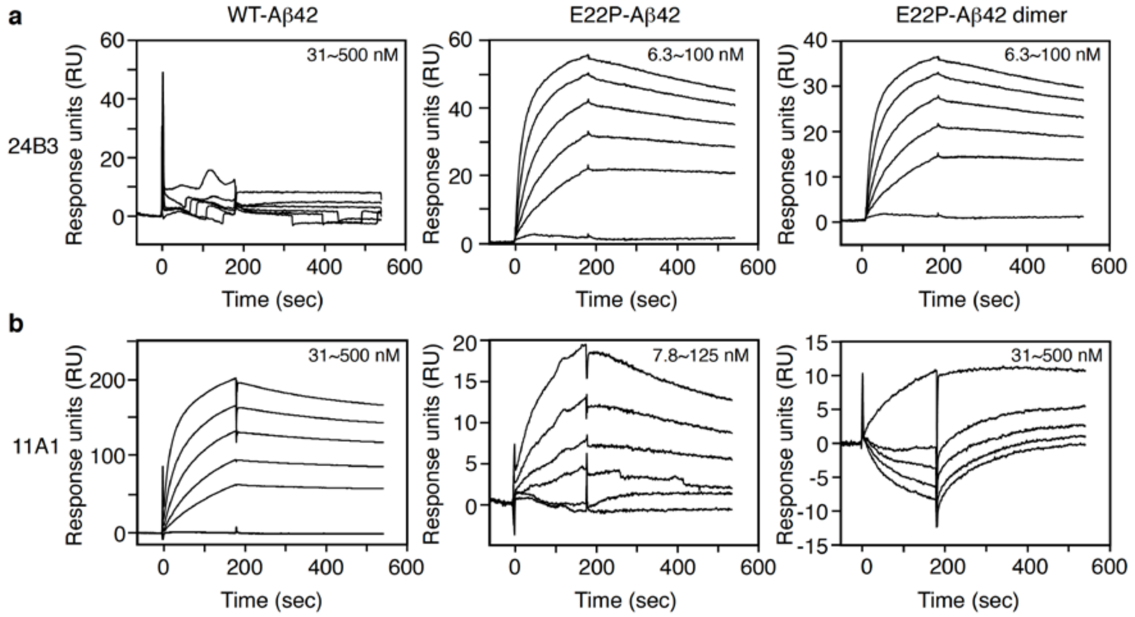

Supplementary Figure 4. SPR sensorgram of 24B3 and 11A1 with Aβ derivatives. The kinetics parameters (K_D, k_a, k_d) of bindings of 24B3 and 11A1 were calculated in Table 1 based on SPR analysis; (**a**) 24B3 (Aβ42 for 31-500 nM, E22P-Aβ42 for 6.3-100 nM, the E22P-Aβ42 dimer for 6.3-100 nM) and (**b**) 11A1 (Aβ42 for 31-500 nM, E22P-Aβ42 for 7.8-125 nM, the E22P-Aβ42 dimer for 31-500 nM).
